# Awareness of Duchenne muscular dystrophy among medical staff in China: a multicenter cross-sectional survey

**DOI:** 10.3389/fped.2026.1735961

**Published:** 2026-05-28

**Authors:** Chun Zhai, Jun Wang, Bing Xia, Ying Guo, Yi Zhang, Yu Jiao, Xiao Juan Shi, Hui Li, Hao Zhou

**Affiliations:** 1 Department of Rehabilitation, National Children’s Medical Center, Children’s Hospital of Fudan University, Shanghai, China; 2 Department of Rehabilitation, The Third Affiliated Hospital of Zhengzhou University, Zhengzhou, China; 3 Department of Rehabilitation, The Northwest Women's and Children's Hospital, Xi'an, Shaanxi, China; 4 Department of Clinical Epidemiology and Clinical Trial Unit, National Children’s Medical Center, Children’s Hospital of Fudan University, Shanghai, China

**Keywords:** awareness, China, Duchenne muscular dystrophy, medical staff, rare disease

## Abstract

**Introduction:**

Although Duchenne muscular dystrophy (DMD) necessitates lifelong integrated management, awareness of the condition among healthcare professionals in China remains lacking. This study assesses DMD-related knowledge within a national rare disease alliance to identify critical gaps in clinical practice.

**Methods:**

Data were collected via an online survey of medical staff across general, maternal-child, and pediatric hospitals. A structured questionnaire, developed by a multicenter expert team and refined through pilot testing, was used to assess awareness of disease diagnosis, treatment, and policy. Convenience sampling was employed, and eligible participants completed the survey anonymously.

**Results:**

Out of 510 distributed questionnaires, 496 valid responses were analyzed. Participants' age ranged from 21 to 68 years, with a mean of 35.73 ± 7.32 years. The overall awareness rate for DMD was 54.64%, with specific rates of 82.46% for treatment, 67.74% for diagnosis, and 42.74% for policy. Univariable analyses indicated that sex, age, occupation, professional title, educational background, professional specialty, and hospital type were associated with awareness levels (*P* < 0.05). Multivariable logistic regression identified occupation, education, and years of experience as independent predictors. Compared with nurses, medical technicians (OR = 2.69, 95% CI 1.39–5.21) and physicians (OR = 2.24, 95% CI 1.15–4.37) demonstrated higher awareness. Notably, participants with postgraduate education exhibited significantly higher awareness than those with undergraduate education (OR = 1.88, 95% CI 1.01–3.53), and practitioners with 5–10 years of experience outperformed those with fewer than 5 years (OR = 2.54, 95% CI 1.16–5.59).

**Conclusion:**

Frontline medical staff in China have critical knowledge gaps regarding DMD genomic diagnostics and related orphan drug policies. These gaps are strongly driven by occupational disparities, especially the systematically lower awareness among nursing staff. To build the multidisciplinary clinical infrastructure needed for the coming era of precision genomic therapy, it is imperative to shift from general professional education to targeted, competency-based DMD training.

## 1 Introduction

Duchenne muscular dystrophy (DMD) is a severe X-linked recessive disorder historically characterized by relentless myofiber degeneration and early mortality (1, 2). However, the contemporary therapeutic landscape, anchored by optimized corticosteroid regimens, non-invasive ventilation, and emerging mutation-specific interventions, has altered the disease's natural history, extending median survival into the third or fourth decade (3–5). Following its formal inclusion in China's First Official List of Rare Diseases (6), this increased longevity shifts the perception of DMD from an exclusively pediatric condition to a chronic lifelong condition, necessitating an integrated clinical management paradigm that incorporates structured adult transitional care and multidisciplinary support.

Translating these therapeutic advances into actual survival benefits hinges on early diagnosis and timely intervention (7). In China, however, this process is critically delayed by diagnostic unfamiliarity among frontline clinicians (8–13). While international registries report a mean diagnostic age of 5 years (14), Chinese patients typically remain undiagnosed until after age eight (15). This lag initiates a prolonged “diagnostic odyssey”: up to 73% of Chinese patients with rare diseases experience initial misdiagnoses, enduring a mean of 4.3 years and three hospital transfers before a definitive diagnosis (16), which incurs substantial financial toxicity from cross-provincial healthcare seeking (17). Consequently, nearly 78.8% of patients miss optimal therapeutic windows or timely specialist referrals (18).

As DMD management evolves toward a lifespan approach, subspecialty diagnostic vigilance must expand beyond pediatric neurology into comprehensive adult-care networks. Despite this need, empirical data quantifying DMD awareness across the Chinese healthcare system remain scarce. To address this gap, we conducted a multicenter survey across general, maternal-child, and pediatric hospitals within a national rare disease medical alliance. By systematically evaluating knowledge gaps in diagnostic, therapeutic, and policy domains, this study provides an evidence base for designing targeted continuous medical education (CME). Addressing these distinct professional blind spots is essential to accelerating early detection, standardizing multidisciplinary transition protocols, and facilitating the implementation of national DMD guidelines.

## 2 Methods

### 2.1 Study participants and eligibility criteria

To assess DMD awareness, a structured questionnaire was distributed via unique QR codes to 31 member hospitals within a national rare disease medical alliance using convenience sampling. Valid responses were obtained from 29 institutions across 21 Chinese provinces and municipalities (institutional response rate: 93.5%).

#### 2.1.1 Target population

The study population comprised three professional cohorts essential to DMD multidisciplinary care: (1) Physicians (pediatricians, neurologists, orthopedists, nutritionists, psychologists, cardiologists, and rehabilitation specialists) responsible for diagnostic evaluation and disease-modifying interventions; (2) Nurses delivering routine inpatient and ambulatory care; and (3) Medical Technicians. Within the Chinese healthcare framework, this latter category inherently integrates allied health professionals, specifically physical (PT), occupational (OT), and speech-language therapists (ST), with diagnostic laboratory and electrophysiology technicians.

#### 2.1.2 Eligibility criteria

Eligibility required a valid clinical license, active employment, and informed consent. *A priori* exclusion criteria for enrollment included: (1) medical trainees lacking independent prescriptive or practice privileges; and (2) non-clinical administrative or logistical personnel. Separately, submissions containing incomplete questionnaire data were excluded prior to statistical analysis.

### 2.2 Ethical approval and consent

This study was approved by the Institutional Review Board of the Children's Hospital of Fudan University [Approval No. 2025(203)]. Informed consent was obtained from all participating healthcare professionals.

### 2.3 Questionnaire design

A structured questionnaire was developed by a multicenter research team, including two pediatric neurologists, six pediatric rehabilitation physicians and therapists, three clinical geneticists, and two rare disease specialists from the Children's Hospital of Fudan University, Henan Maternal and Child Health Hospital, and Northwest Women's and Children's Hospital. The questionnaire was based on the dichotomous framework established by Krosnick (19), as well as the Chinese Guidelines for the Diagnosis and Treatment of Rare Diseases (2019 edition) and current domestic consensus documents (20, 21).

A series of structured online discussions was conducted via teleconference to refine the instrument, resulting in a preliminary version comprising 13 core statements. A pilot study (*n* = 90) was conducted across three participating hospitals via convenience sampling to evaluate the questionnaire's diagnostic accuracy, clinical applicability, and wording clarity. Psychometric analysis demonstrated satisfactory internal consistency (Cronbach's alpha = 0.87) and construct validity, supported by a Kaiser-Meyer-Olkin (KMO) index of 0.835. The final version consisted of two sections:

1. Demographic characteristics: age, occupation, professional title, educational background, years of work experience, specialty, hospital type and grade, and geographic region.
2. Questionnaire content: The instrument comprised 13 statements (Table 1) organized into diagnostic (Items 1–5), therapeutic (Items 6–10), and policy knowledge domains (Items 11–13). Each statement was rated dichotomously (“Known” vs. “Unknown”).

**Table 1:** Factual statements used in the structured questionnaire to assess DMD awareness.

Diagnostic section
1: Duchenne muscular dystrophy (DMD) is an X-linked recessive disorder
2: Multiplex ligation-dependent probe amplification (MLPA) is the main genetic test for DMD
3: Elevated creatine kinase levels are characteristic of DMD
4: Gastrocnemius hypertrophy is a common sign in children with DMD
5: waddling gait is typical in children with DMD

Treatment section
6: DMD is a multisystemic disorder involving various physiological systems
7: Patients with DMD should undergo regular monitoring of cardiopulmonary function
8: DMD may present with cognitive, linguistic, and swallowing impairments in addition to motor limitations.
9: DMD requires early, standardized, and long-term rehabilitation assessment and intervention
10: Corticosteroid therapy can slow disease progression in children with DMD

Policy section
11: Gene-modifying therapies for DMD are emerging in clinical practice
12: DMD is included in China's official National Rare Disease Catalogue
13: Comprehensive guidelines for DMD care have been published both domestically and internationally

### 2.4 Data collection

The survey was established using the online platform Wenjuanxing (https://www.wjx.cn), a secure tool that generates unique QR-coded access links for respondents. Digital survey identifiers were distributed through the Children's Hospital of Fudan University medical alliance. Designated institutional contacts disseminated the survey via email to their respective workgroups. Between March 23 and 30, 2025, eligible participants completed the electronic questionnaire by scanning a provided QR code with their mobile devices.

### 2.5 Statistical analysis

Statistical analyses were performed using SPSS version 25.0. Continuous variables were expressed as mean ± standard deviation or median (range) and compared using Student's *t*-tests or Mann–Whitney *U*-tests. Categorical variables were presented as frequencies (percentages) and evaluated using R × C Pearson's Chi-square tests. Following modified Bloom's cut-offs (22), awareness was stratified into three tiers: high (≥80%, 11–13 items), moderate (60%–79%, 8–10 items), and low (<60%, 0–7 items) tiers. Significant multi-categorical variations were further assessed via *post hoc* Chi-square partitioning with adjusted thresholds (e.g., *α*_adj_ = 0.0125). To identify independent predictors of baseline clinical competency, variables with *P* < 0.05 in univariable analysis were incorporated into a multivariable logistic regression model. Based on the Mastery Learning framework (23), the outcome was dichotomized into high (≥80%) vs. moderate/low (<80%) awareness. Results were reported as odds ratios (OR) with 95% confidence intervals (CI). Statistical significance was defined as a two-tailed *P* < 0.05.

## 3 Results

### 3.1 Demographic characteristics

Out of 510 distributed surveys, 496 (97.25%) valid responses were analyzed following the exclusion of 14 incomplete submissions. The study population exhibited a mean age of 35.73 ± 7.32 years (range: 21–68) and was predominantly female (71.98%; *n* = 357), spanning 21 Chinese provinces and municipalities. The demographic characteristics of the study cohort are detailed in Table 2.

**Table 2:** Demographic characteristics of the study population (*N* = 496).

Characteristics, *n* (%)	Summary, *n* (%)
Total	496 (100)
Mean age ± SD	35.73 ± 7.32
Sex	
Male	139 (28.02)
Female	357 (71.98)
Age	
21–29	102 (20.56)
30–39	262 (52.82)
40–49	106 (21.37)
50 and above	26 (5.24)
Occupation	
Nurse	109 (21.98)
Medtech	205 (41.33)
Doctor	182 (36.69)
Professional title	
junior	185 (37.30)
Intermediate	227 (45.77)
Senior	84 (16.94)
Education level	
Undergraduate or below	367 (73.99)
Master's degree or above	129 (26.01)
Years of working	
<5 years	74 (14.92)
5–10 years	164 (33.06)
10–20 years	200 (40.32)
>20 years	58 (11.69)
Professional specialty	
Rehabilitation	267 (53.83)
Non-rehabilitation	229 (46.17)
Hospital type	
General Hospital	245 (49.40)
Children's Hospital	86 (17.34)
Maternal and Child Health Hospital	165 (33.27)
Hospital level	
Tertiary hospital	412 (83.06)
Secondary hospital	84 (16.94)
Geographical region of hospital	
Eastern	107 (21.57)
Western	114 (22.98)
Southern	86 (17.34)
Northern	29 (5.85)
Central	160 (32.26)

Continuous variables are presented as mean ± SD or median (range), and categorical variables are listed as *n* (%). Medtech, Medical technicians.

**Figure 1:**
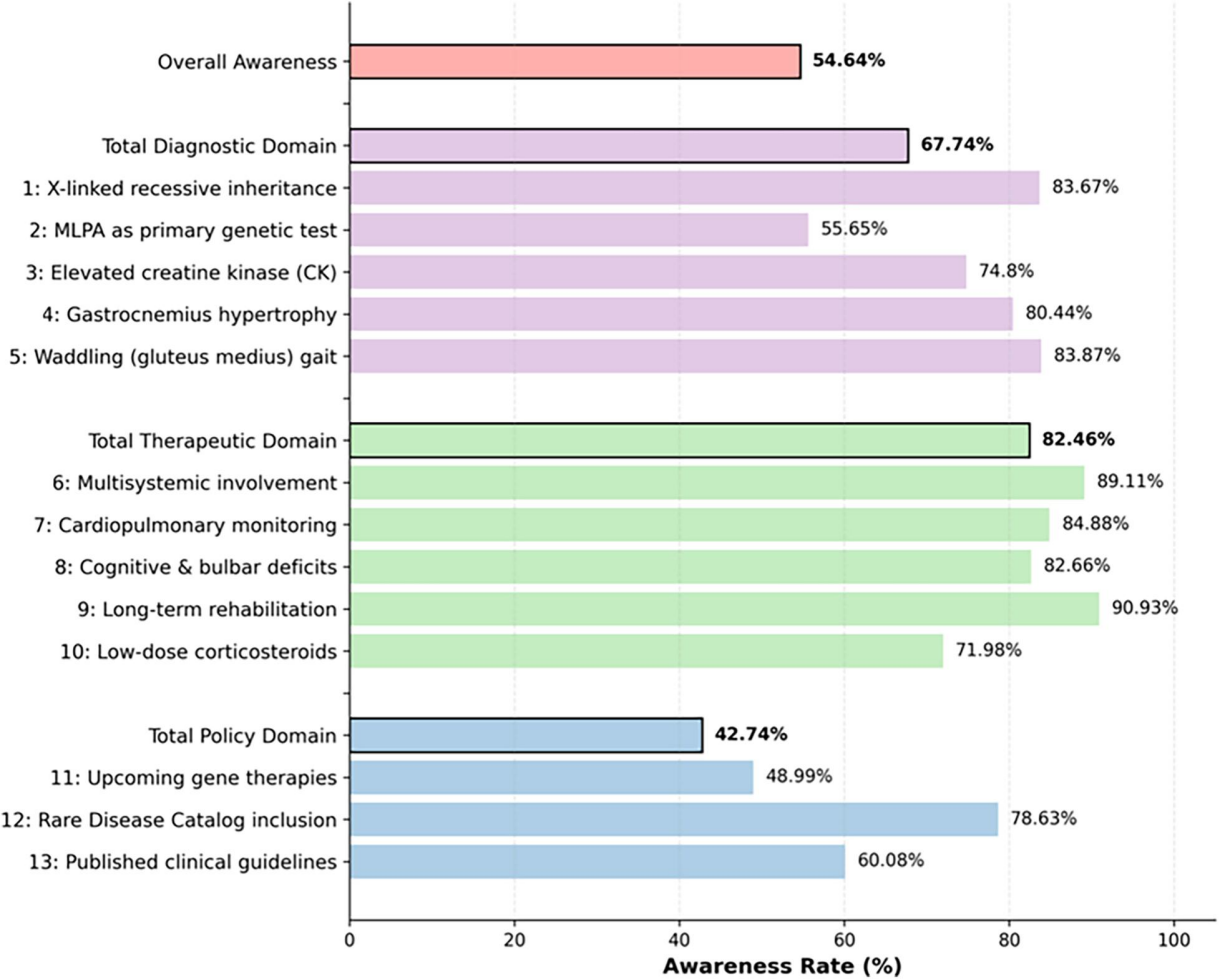
Awareness rates of Duchenne muscular dystrophy across distinct clinical domains.

Figure 1. Percentage of correct responses across questionnaire items. *Y*-axis labels are abbreviated as clinical keywords (Items 1–13); refer to Table 1 for the full verbatim statements.

### 3.2 Awareness of DMD among medical staff

The overall DMD awareness was 54.64% (*n* = 271; Figure 1). Domain-specific awareness was highest for treatment (82.46%, *n* = 409), followed by diagnosis (67.74%, *n* = 336) and policy (42.74%, *n* = 212). Item-level analysis revealed notable intra-domain variation. Diagnostic awareness (range: 55.65%–83.87%) was highest for recognizing waddling gait (83.87%) and lowest for identifying MLPA as the primary genetic test (55.65%). Treatment awareness (71.98%–90.93%) peaked for acknowledging long-term rehabilitation needs (90.93%), whereas low-dose corticosteroid efficacy was least recognized (71.98%). Finally, policy knowledge (48.99%–78.63%) was highest for DMD's inclusion in the national Rare Disease Catalog (78.63%) and lowest for upcoming clinical gene therapies (48.99%).

### 3.3 Comparison of demographic characteristics between the DMD awareness groups

Of the 496 participants, 54.64% (*n* = 271) were classified as the high-awareness group (≥80% correct on 11–13 items). The remaining cohort was stratified into moderate (60%–79%, 8–10 items) and low (<60%, 0–7 items) awareness groups, comprising 25.81% (*n* = 128) and 19.56% (*n* = 97), respectively. The demographic distribution across these three tiers is detailed in Table 3.

**Table 3:** Comparison of overall awareness rates among different demographic characteristics.

Characteristics	Low awareness	Moderate awareness	High awareness	χ*^2^*	*P*-value
Total, *n* (%)	97 (19.6)	128 (25.8)	271 (54.6)		
Sex, *n* (%)					
Male	24 (17.3)	27 (19.4)	88 (63.3)	6.22	**0**.045*
Female	73 (20.4)	101 (28.3)	183 (51.3)		
Age, *n* (%)					
21–29	20 (19.6)	35 (34.3)	47 (46.1)	13.76	**0** **.** **032** *
30–39	59 (22.5)	67 (25.6)	136 (51.9)		
40–49	15 (14.2)	20 (18.9)	71 (67.0)		
50 and above	3 (11.5)	6 (23.1)	17 (65.4)		
Occupation, *n* (%)					
Nurse	43 (39.4)	29 (26.6)	37 (33.9)	45.52	**<0**.001***
Medtech	32 (15.6)	61 (29.8)	112 (54.6)		
Doctor	22 (12.1)	38 (20.9)	122 (67.0)		
Professional Title, *n* (%)					
Junior	47 (25.4)	52 (28.1)	86 (46.5)	12.93	0.012*
Intermediate	41 (18.1)	58 (25.6)	128 (56.4)		
Senior	9 (10.7)	18 (21.4)	57 (67.9)		
Education Level, *n* (%)					
Undergraduate or below	81 (22.1)	106 (28.9)	180 (49.1)	17.81	**<0**.001***
Master's degree or above	16 (12.4)	22 (17.1)	91 (70.5)		
Years of working, *n* (%)					
<5 years	15 (20.3)	29 (39.2)	30 (40.5)	11.09	0.086
5–10 years	35 (21.3)	38 (23.2)	91 (55.5)		
10–20 years	39 (19.5)	48 (24.0)	113 (56.5)		
>20 years	8 (13.8)	13 (22.4)	37 (63.8)		
Professional specialty, *n* (%)					
Rehabilitation	39 (14.6)	79 (29.6)	149 (55.8)	10.59	**0**.005**
Non-rehabilitation	58 (25.3)	49 (21.4)	122 (53.3)		
Hospital type, *n* (%)					
General Hospital	42 (17.1)	74 (30.2)	129 (52.65)	12.44	**0** **.** **014** *
Children's Hospital	11 (12.8)	20 (23.3)	55 (63.95)		
MCH hospital	44 (26.7)	34 (20.6)	87 (52.73)		
Hospital level, *n* (%)					
Tertiary hospital	85 (20.6)	104 (25.2)	223 (54.13)	1.85	0.396
Secondary hospital	12 (14.3)	24 (28.6)	48 (57.14)		
Geographical region, *n* (%)					
Eastern	18 (16.8)	23 (21.5)	66 (61.68)	11.12	0.195
Western	24 (21.1)	24 (21.1)	66 (57.89)		
Southern	22 (25.6)	29 (33.7)	35 (40.70)		
Northern	5 (17.2)	9 (31.0)	15 (51.72)		
Central	28 (17.5)	43 (26.9)	89 (55.63)		

Categorical variables are presented as n (%). Bold *P* values indicate statistically significant differences in the distribution of overall awareness levels across subgroups, as assessed by the χ² test (*P* < 0.05).

* *P* < 0.05.

** *P* < 0.01.

*** *P* < 0.001.

Medtech, medical technicians; MCH hospital, Maternal and Child Health Hospital.

### 3.4 Factors associated with awareness

Univariable analyses (Table 3) showed significant differences across the three awareness tiers regarding sex (*P* = 0.045), age (*P* = 0.032), occupation (*P* < 0.001), professional title (*P* = 0.012), educational level (*P* < 0.001), professional specialty (*P* = 0.005), and hospital type (*P* = 0.014). In *post hoc* pairwise comparisons (*α*_adj_ = 0.0125), occupation was the only factor demonstrating significant differences across all tier combinations (low vs. moderate, *P* = 0.002; moderate vs. high, *P* = 0.006; low vs. high, *P* < 0.001). Educational level differed between the high tier and both lower tiers (vs. low, *P* = 0.002; vs. moderate, *P* = 0.001), with no difference between the low and moderate cohorts (*P* = 1.000). Significant differences in professional specialty were limited to the low vs. moderate-awareness groups comparison (*P* = 0.002), whereas professional title differed only between the low- and high-awareness groups (*P* = 0.003). After adjustment, pairwise comparisons for hospital type (minimum *P* = 0.014), sex, and age were not statistically significant.

Multivariate logistic regression analysis (Table 4) identified occupation, educational attainment, and years of professional experience as significant independent predictors of overall DMD awareness among healthcare professionals. Compared with nurses, medical technicians (OR = 2.69, 95% CI 1.39–5.21) and physicians (OR = 2.24, 95% CI 1.15–4.37) exhibited significantly higher awareness. Participants with a master's degree or higher demonstrated greater awareness than those with a bachelor's degree (OR = 1.88, 95% CI 1.01–3.53). Furthermore, individuals with 5–10 years of work experience showed significantly higher awareness compared with those with less than 5 years of experience (OR = 2.54, 95% CI 1.16–5.59).

**Table 4:** Univariate and multivariate logistic regression analyses of overall knowledge.

Variables	Univariable analysis		Multivariate analysis	
	OR (95% CI)	*P*-value	OR (95% CI)	*P*-value
Sex, *n* (%)				
Male	1.00		1.00	
Female	0.61 (0.41–0.91)	**0** **.** **016** *	0.89 (0.56-1.42)	0.631
Age, *n* (%)				
21–29	Reference		Reference	
30–39	1.26 (0.80–2.00)	0.318	0.60 (0.28–1.29)	0.190
40–49	2.37 (1.35–4.16)	**0** **.** **003** **	1.07 (0.39–2.92)	0.898
50 and above	2.21 (0.90–5.42)	0.083	1.12 (0.26–4.85)	0.880
Occupation, *n* (%)				
Nurse	Reference		Reference	
Medtech	2.34 (1.45–3.80)	**0** **.** **001** **	2.69 (1.39–5.21)	**0** **.** **004** **
Doctor	3.96 (2.39–6.54)	**<0** **.** **001** ***	2.24 (1.15–4.37)	**0** **.** **018** *
Professional Title, *n* (%)				
Junior	Reference		Reference	
Intermediate	1.49 (1.01–2.20)	**0** **.** **046** *	1.35 (0.79–2.32)	0.278
Senior	2.43 (1.41–4.18)	**0** **.** **001** **	1.13 (0.48–2.69)	0.777
Education Level, *n* (%)				
Undergraduate or below	Reference		Reference	
Master's degree or above	2.49 (1.62–3.83)	**<0** **.** **001** ***	1.88 (1.01–3.53)	**0** **.** **048** *
Years of Working, *n* (%)				
<5 years	Reference		Reference	
5–10 years	1.83 (1.05–3.19)	**0** **.** **034** *	2.54 (1.16–5.59)	**0** **.** **020** *
10–20 years	1.90 (1.11–3.27)	**0** **.** **020** *	2.13 (0.83–5.43)	0.114
>20 years	2.58 (1.27–5.25)	**0** **.** **009** **	2.32 (0.61–8.83)	0.219
Geographical region, *n* (%)				
Eastern	Reference		Reference	
Western	0.85 (0.50–1.46)	0.566	0.99 (0.52–1.87)	0.968
Southern	0.43 (0.24–0.76)	**0** **.** **004** **	0.60 (0.32–1.14)	0.117
Northern	0.67 (0.29–1.52)	0.334	0.73 (0.30–1.79)	0.494
Central	0.78 (0.47–1.28)	0.326	0.89 (0.50–1.59)	0.684

ORs are presented with 95% CIs. Bold *P*-value indicate statistically significant associations with overall knowledge in the corresponding univariable or multivariable logistic regression model (*P* < 0.05).

* *P* < 0.05

** *P* < 0.01

*** *P* < 0.001

Medtech, medical technicians.

### 3.5 Subgroup exploratory analysis

**Figure 2:**
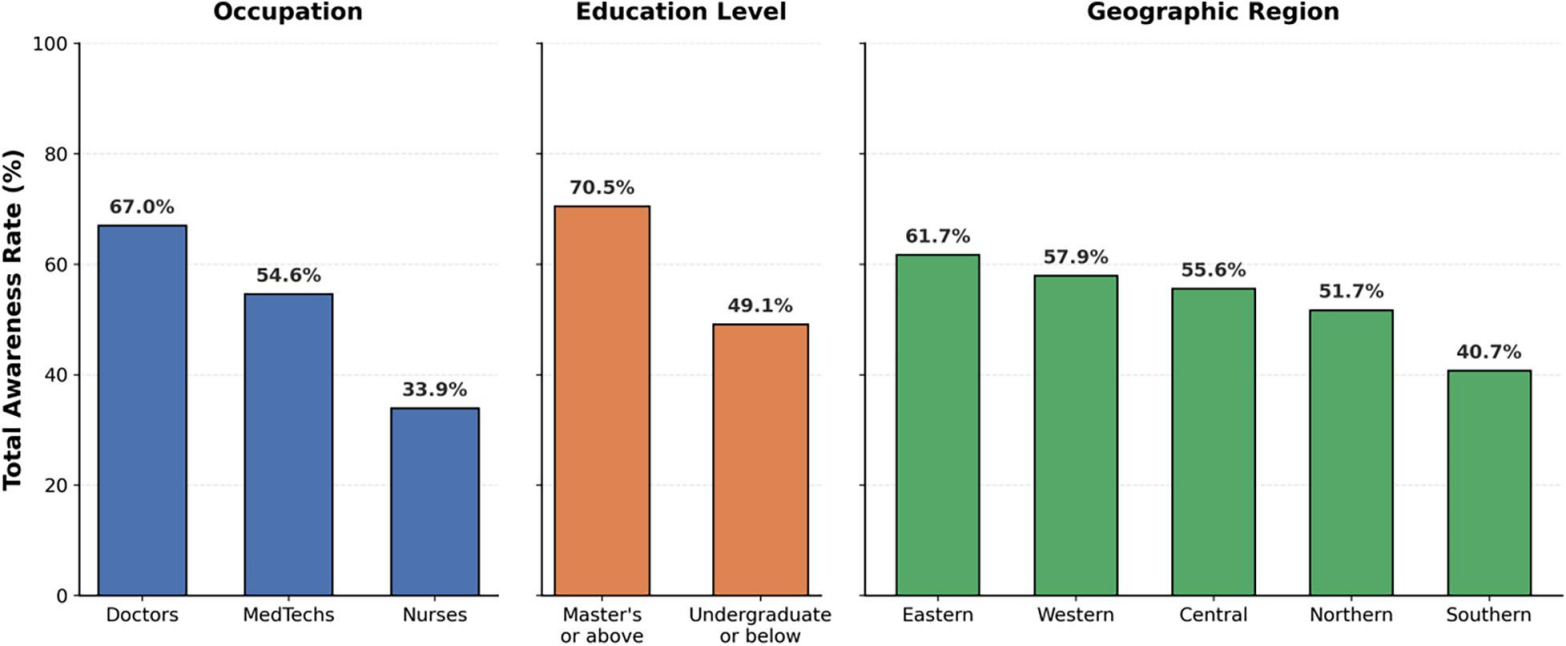
Subgroup exploratory analysis of total awareness rates.

To further delineate the distribution of clinical competency, an exploratory subgroup analysis evaluated the proportion of participants achieving high awareness (≥80%) across key demographic categories (Figure 2). Occupationally, this proportion was highest among physicians (67.0%), followed by medical technicians (54.6%) and nurses (33.9%). Educationally, participants with a master's degree or above outperformed those with an undergraduate degree or below (70.5% vs. 49.1%). Geographically, high awareness peaked in the Eastern region (61.7%), sequentially declining across the Western (57.9%), Central (55.6%), Northern (51.7%), and Southern (40.7%) regions.

## 4 Discussion

Our assessment showed that 54.64% of Chinese medical staff achieved high DMD awareness (≥80%), meeting the threshold for baseline clinical competency. Stratifying the remaining cohort into moderate (60%–79%) and low (<60%) awareness tiers provided critical clinical granularity. Practitioners with moderate awareness recognized basic symptoms but had substantial knowledge gaps regarding genomic diagnostics and orphan drug policies. Conversely, the low-awareness cohort lacked fundamental disease recognition, which poses a major risk for initial misdiagnosis and delayed referrals. This three-tier distribution is consistent with previously reported global bottlenecks in rare disease education (24, 25). Collectively, these stratified knowledge deficits indicate that standard medical curricula struggle to keep pace with rapid translational advances, reflecting the urgent need for targeted, competency-based continuing medical education.

Domain-specific analyses revealed a significant gap between available medical infrastructure and frontline clinical practice. Specifically, only 55.65% of participants recognized MLPA as the definitive diagnostic tool. This lack of recognition likely contributes to prolonged diagnostic delays in Chinese rare disease cohorts (12–16). Although MLPA has been nationally promoted since 2015 (26), this knowledge gap persists despite the expansion of next-generation sequencing and AI-assisted imaging (27). As a result, pediatric patients may experience avoidable diagnostic delays, irreversible motor deterioration, and unnecessary invasive procedures such as empirical muscle biopsies prior to genetic confirmation. From a therapeutic perspective, domestic guidelines (20, 21) have effectively standardized physical rehabilitation. Nevertheless, the understanding of continuous glucocorticoid therapy remains suboptimal (71.98%). This gap is particularly concerning as China advances clinical trials of emerging therapies such as CRISPR-based interventions (28) and exon-skipping approaches (29). Clinicians who are hesitant to adopt established steroid regimens may face additional challenges in safely managing the immunological complexity and adverse effects associated with novel genomic therapies. In addition, limited awareness of (42.74%) actively constrains the clinical impact of recent regulatory advancements. Since DMD was included on the 2018 National Rare Disease List, 95 orphan drugs have been added to the 2024 national insurance catalog (30, 31). However, these reimbursement mechanisms are ineffective if prescribing physicians remain unaware of them. This policy ignorance restricts patient access to essential therapeutics, ultimately exacerbating inequities in lifelong DMD management.

In the present study, multivariable analysis identified educational attainment, clinical experience, and occupation as independent predictors. Advanced education significantly predicted high awareness (Figure 2), likely reflecting greater knowledge of molecular genetics (32) and orphan drug literature. Nonetheless, education level did not differentiate between the low and moderate awareness tiers (*P* = 1.000). Regarding clinical experience, senior staff (>20 years) initially showed the highest univariable odds of high awareness (OR = 2.58). Yet, in multivariable analysis, mid-career professionals (5–10 years; OR = 2.54) emerged as the primary independent predictor, overriding age and job title. Therefore, while senior clinicians maintain awareness through empirical exposure, mid-career practitioners successfully combine recent genomic education with frontline clinical practice.

At the institutional level, professional specialty was the key factor distinguishing low from moderate awareness. Severe knowledge deficits (<60%) were nearly twice as common among non-rehabilitation staff (25.3% vs. 14.6%). Because initial DMD symptoms often present to general pediatrics or orthopedics, diagnostic vigilance outside specialized neuromuscular units is critical. Although statistical adjustment (*α*_adj_ = 0.0125) rendered hospital type non-significant, descriptive data showed higher low-awareness rates in Maternal and Child Health (MCH) hospitals (26.7%) than in specialized Children's hospitals (12.8%). Given that MCH hospitals frequently serve as the first point of contact for early developmental concerns, this represents a clinically important gap. Occupationally, nurses consistently exhibited lower awareness across all tiers. While the proficiency of physicians and technicians aligns with their diagnostic roles, the lower awareness among nurses indicates a structural gap (Figure 2). Outside primary diagnostic decision-making, nurses receive less exposure to advances in genomics. This is in line with reported under-preparation in rare disease management (33).

This occupational gap also accounted for the observed regional variations. Geographic region was not significant in the three-tier univariable analysis (*P* = 0.195). However, evaluating only the high-awareness threshold (≥80%) revealed a lower proficiency rate in the Southern cohort (Figure 2) compared with the Eastern (61.7%) and Western (57.9%) regions. Importantly, geography remained non-significant in the multivariable regression (Table 4). Cross-tabulation (Supplementary Appendix 1) revealed the underlying cause: nurses comprised 41.9% (*n* = 36/86) of the Southern cohort, nearly double the overall average (22.0%). Because nurses consistently scored lower, this large proportion of nurses artificially lowered the Southern region's overall score. Thus, the perceived geographic disparity is primarily an artifact of the occupational composition.

These nursing deficits reflect systemic constraints within China's Duchenne care continuum. The traditional physician-led, outpatient-centric model isolates ward nurses from the rare-disease diagnostic loop, thereby restricting longitudinal clinical exposure. To align with international standards (34), medical education must transition from generic training to stratified, competency-based frameworks. For nurses, this requires targeted pathways: an acute-care module for general inpatient nurses (focusing on perioperative airway clearance and stress-dose corticosteroids) and a care-coordination module that cultivates specialized neuromuscular nurses for cardiopulmonary monitoring and psychosocial triage. Integrating these curricula into simulation-based training (35) and national networks will standardize baseline proficiencies. Ultimately, formally incorporating specialized nursing roles into regional DMD centers, guided by TREAT-NMD pathways (36), will optimize multidisciplinary team (MDT) management (37, 38) and establish the infrastructure necessary for emerging precision genomic therapies.

Several methodological limitations of this study must be acknowledged. First, our convenience sampling strategy inherently skewed the cohort toward practitioners in well-resourced tertiary centers. Consequently, extrapolating these findings likely overestimates the baseline diagnostic readiness of primary and rural healthcare settings, which are typically the actual sites where initial diagnostic delays originate. Future research should employ stratified random sampling to assess knowledge gaps across all healthcare tiers accurately. Second, self-administered assessments carry inherent risks of social desirability bias. Driven by normative professional expectations, clinicians may overestimate their clinical proficiency (39, 40). Therefore, the reported 54.64% awareness rate should be pragmatically interpreted as an optimistic upper limit. Rather than weakening our conclusions, this potential performance inflation suggests that true frontline knowledge deficits are likely more severe, directly reinforcing the clinical necessity of the targeted educational interventions proposed herein. To overcome the intrinsic limitations of subjective self-reporting, subsequent competency research must transition toward objective evaluations. Incorporating objective structured clinical examinations (OSCEs) or high-fidelity simulated clinical vignettes will be crucial for accurately quantifying cognitive deficits and rigorously monitoring the efficacy of future training programs.

## 5 Conclusion

Chinese frontline medical staff demonstrate substantial gaps in knowledge of DMD genomic diagnostics and healthcare policy. Our findings indicate that these gaps are driven primarily by occupational disparities, specifically the peripheral role of nursing staff in diagnostic workflows. To reduce initial diagnostic delays and establish the multidisciplinary infrastructure necessary for emerging precision genomic therapies, continuing medical education must transition from generic curricula to targeted, competency-based frameworks. Strengthening diagnostic literacy and pharmacological awareness among nurses and transitional care teams is essential for improving lifelong DMD management.

## Data Availability

The raw data supporting the conclusions of this article will be made available by the authors, without undue reservation.
