# Supplementary figures and images for: Awareness of Duchenne muscular dystrophy among medical staff in China: a multicenter cross-sectional survey
Source: Front Pediatr. 2026 May 28;14:1735961. doi: 10.3389/fped.2026.1735961 (PMC13253694; doi:10.3389/fped.2026.1735961)

Appendix 1: Occupational Composition Across Geographic Regions

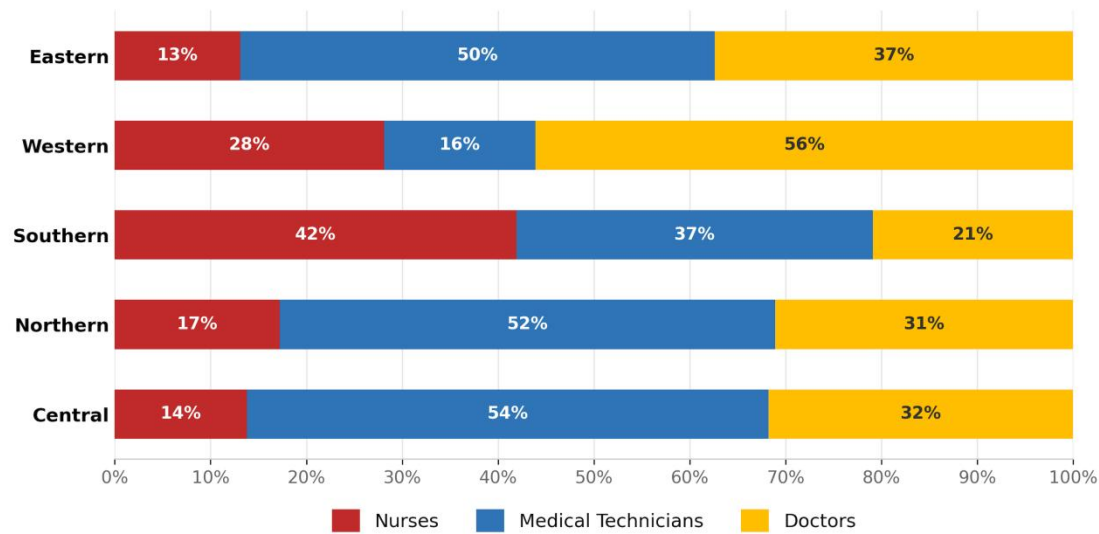

Supplement: Supplementary file 1 [file Datasheet1.pdf]
